# A prediction model for acute respiratory distress syndrome in immunocompetent adults with adenovirus-associated Pneumonia: a multicenter retrospective analysis

**DOI:** 10.1186/s12890-023-02742-8

**Published:** 2023-11-06

**Authors:** Fengyu Lin, Qianhui Zhou, Wen Li, Wenchao Xiao, Sha Li, Ben Liu, Haitao Li, Yanhui Cui, Rongli Lu, Yi Li, Yan Zhang, Pinhua Pan

**Affiliations:** 1 Department of Respiratory Medicine, Branch of National Clinical Research Center for Respiratory Disease, Xiangya Hospital, National Key Clinical Specialty, Central South University, Changsha, China; 2 Center of Respiratory Medicine, Xiangya Hospital, Central South University, Changsha, Hunan 410008 China; 3 Hunan Engineering Research Center for Intelligent Diagnosis and Treatment of Respiratory Disease, Changsha, China; 4 National Clinical Research Center for Geriatric Disorders, Xiangya Hospital, Changsha, China; 5 Clinical Research Center for Respiratory Diseases in Hunan Province, Changsha, China; 6 Department of Respiratory Medicine, Zhuzhou Central Hospital, Zhuzhou, China; 7 Department of Cardiology, Yiyang Central Hospital, Yiyang, China; 8 Department of Radiology, Xiangya Hospital, Central South University, Changsha, China; 9 Department of Emergency Medicine, Xiangya Hospital, Central South University, Changsha, China; 10 First Department of Thoracic Medicine, The Affiliated Cancer Hospital of Xiangya School of Medicine, Hunan Cancer Hospital, Central South University, Changsha, China; 11 Furong Laboratory, Changsha, China

**Keywords:** Adenovirus Pneumonia, Acute respiratory distress syndrome, HAdVs, ARDS, Severe HAdV Infection, HAdV-related ARDS

## Abstract

**Background:**

In recent years, the number of human adenovirus (HAdV)-related pneumonia cases has increased in immunocompetent adults. Acute respiratory distress syndrome (ARDS) in these patients is the predominant cause of HADV-associated fatality rates. This study aimed to identify early risk factors to predict early HAdV-related ARDS.

**Methods:**

Data from immunocompetent adults with HAdV pneumonia between June 2018 and May 2022 in ten tertiary general hospitals in central China was analyzed retrospectively. Patients were categorized into the ARDS group based on the Berlin definition. The prediction model of HAdV-related ARDS was developed using multivariate stepwise logistic regression and visualized using a nomogram.

**Results:**

Of 102 patients with adenovirus pneumonia, 41 (40.2%) developed ARDS. Overall, most patients were male (94.1%), the median age was 38.0 years. Multivariate logistic regression showed that dyspnea, SOFA (Sequential Organ Failure Assessment) score, lactate dehydrogenase (LDH) and mechanical ventilation status were independent risk factors for this development, which has a high mortality rate (41.5%). Incorporating these factors, we established a nomogram with good concordance statistics of 0.904 (95% CI 0.844–0.963) which may help to predict early HAdV-related ARDS.

**Conclusion:**

A nomogram with good accuracy in the early prediction of ARDS in patients with HAdV-associated pneumonia may could contribute to the early management and effective treatment of severe HAdV infection.

## Introduction

Human adenoviruses (HAdVs) are a group of double-stranded, non-enveloped DNA viruses that cause acute respiratory infections [1]. In immunocompromised patients and children, HAdVs are one of the most common causes of serious infections, whereas in immunocompetent adults, HAdV infection generally presents as a self-limited infection with mild flu-like symptoms [2–4]. In recent years, the number of serious and sometimes fatal adenovirus infections has increased in immunocompetent adults [5–7]. Similar to other respiratory viral infections, serious adenovirus infections usually present as adenoviral pneumonia in immunocompetent adults [8, 9]. Compared to studies on pediatric or immunocompromised patients, relatively fewer studies have focused on HAdV-associated pneumonia in immunocompetent adults [10].

Previous studies have reported that acute respiratory distress syndrome (ARDS) is one of the most important hallmarks of severe infections and is classically associated with high mortality in viral infections [11–13]. However, no large-sample studies have specifically focused on HAdV-related ARDS hitherto. Occasional single-case reports and small case series have mentioned rapid progression of HAdV infections to ARDS, followed by life-threatening sequelae and death [14–18]. It can be concluded from these reports that there are limited options for treatment of severe HAdV infections, especially HAdV-related ARDS [1]. HAdV-related ARDS also commonly results in complications such as requirement for mechanical ventilation, extracorporeal membrane oxygenation (ECMO), admission to intensive care units (ICU), and can even cause death. These findings suggest that more attention should be paid to HAdV-associated ARDS. A prediction model could assist in early identification of patients at high risk for the development of ARDS to optimize early recognition and treatment. Although ARDS risk prediction models have been previously reported in various populations, such as non-emergency department hospitalized patients [19], patients with severe acute pancreatitis [20], and sepsis patients [21], no prediction models have been proposed to predict HAdV-related ARDS.

In this retrospective study, we have reported the clinical characteristics and epidemiological trends of adenovirus-related pneumonia as well as the clinical characteristics and factors associated with the development of ARDS in Central South China. Moreover, through multivariable logistic regression analysis and the development of a nomogram model, we have constructed an efficient ARDS prediction model for the early identification of the risk of ARDS among patients with adenovirus-related pneumonia. This could contribute to better management and effective therapeutic strategies for severe HAdV infection.

## Methods

### Study design and participants

We retrospectively collected the data of patients who were diagnosed with community-acquired HAdV pneumonia between June 21, 2018, and May 2, 2022 from 10 centers in Central South China. All patients were adults (age ≥ 18 years) and were diagnosed with community-acquired pneumonia [22] at hospital admission. The diagnosis was laboratory-confirmed by the positive detection of human adenovirus in bronchoalveolar lavage fluid (BALF), sputum, or blood, using next-generation sequencing or polymerase chain reaction (PCR) [23, 24]. This was done in accordance with previously established protocols. If available, laboratory-confirmed HAdV typing data were also collected.

All patients underwent routine microbial etiological examination after admission: (i)bacteria and fungi were detected by culture; (ii) *Mycoplasma pneumonia, Influenza A virus, Influenza B virus, Legionella pneumophila*, *Chlamydia pneumoniae*, and *Coxiella burnetii* were identified by serological tests; and (iii) coronaviruses, *Human metapneumovirus*, influenza viruses, parainfluenza viruses, adenoviruses, rhinovirus, *Respiratory syncytial virus*, and enterovirus were tested for by PCR.

The exclusion criteria were as follows: pregnancy, immunocompromised state due to any cause [25], co-infection with bacterial fungi or other respiratory viruses on admission, and missing medical records.

Ultimately, 102 immunocompetent adult patients with human adenovirus pneumonia were included in the final analysis. The included patients who developed ARDS (based on the Berlin definition [26]) during hospitalization were categorized into the ARDS group [27]. This study was approved by the ethics committee of Xiangya Hospital of Central South University (No. 202,104,005). The data were anonymized and the requirement for informed consent was waived.

### Measurements

The collected information included patients’ demographic data, symptoms, vital signs, comorbidities, viral subtype, SOFA (Sequential Organ Failure Assessment) score, APACHE II (Acute Physiology and Chronic Health Evaluation II) score, radiographic characteristics, laboratory findings, oxygen support requirement and therapeutic measures. The above information was collected within the first 24 h of hospital admission. During the entire period of hospitalization, oxygen support requirement, history of therapeutic measures, complications, and clinical outcomes were also collected from medical records. Acute cardiac injury and acute kidney injury were defined according to published criteria [28, 29].

### Statistical analysis

Normally distributed data were expressed as means ± standard deviations, variables with non-normal distribution were reported as medians (interquartile range), and categorical variables were summarized as frequencies and percentages. Continuous data with normal distribution were compared using the Student’s t-test, non-normally distributed variables were tested using the Mann–Whitney U test, and categorical variable rates were assessed using the chi-square test or Fisher’s exact test. Kaplan–Meier curves were created for each group and compared using the log-rank test. Univariate logistic regression analyses were performed to assess risk factors for adenovirus-associated ARDS. The final multivariate prediction model for adenovirus-associated ARDS was developed using multivariate stepwise logistic regression and visualized using a nomogram. The calibration curve, concordance statistic (C-statistic), area under the receiver operating characteristic (AUROC) curves, and decision curve analysis (DCA) were used to assess the model performance. Statistical significance was set at P ≤ 0.05. All probability tests were two tailed. All statistical analyses and graphs were generated using SPSS version 26.0 (IBM Corporation, Armonk, NY, USA), GraphPad Prism version 9.0 software (GraphPad Software Inc., San Diego, CA, USA), or R-4.1.2 software (R Foundation for Statistical Computing, Vienna, Austria).

## Results

### Clinical characteristics

**Fig. 1:**
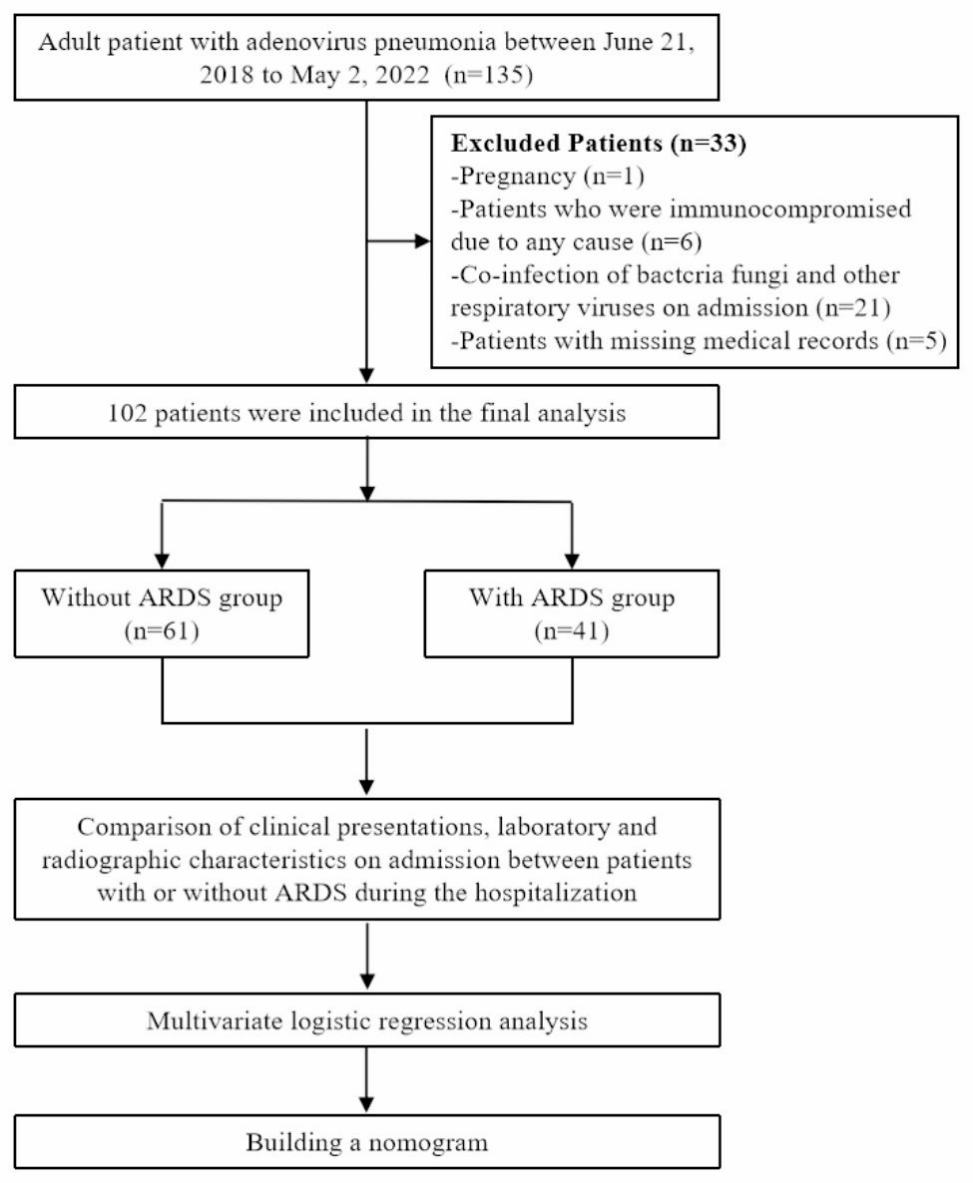
Flow diagram of the study population

In total of 135 patients diagnosed with community-acquired human adenovirus pneumonia were screened. Of these, 33 patients with pregnancy, immunocompromised due to any cause, co-infection of bacteria, fungi, and other respiratory viruses on admission, or with missing medical records were subsequently excluded according to the established exclusion criterion. Finally, 102 immunocompetent adult patients with human adenovirus pneumonia were included in this study. (Fig. 1). The mean age of the patients included was 38.0 years, and 94.1% of them were males (96/102). The most common clinical manifestations were fever (98.0%, 100/102), cough (86.3%, 88/102), sputum production (78.4%, 80/102), dyspnea (55.9%, 57/102), fatigue (52.9%, 54/102), and moist rales (68.6%, 70/102). For comorbidities, twelve patients (11.8%) had hypertension, six patients (5.9%) had diabetes, nine patients (8.8%) had chronic lung disease, eight patients (7.8%) had chronic liver diseases, eight patients (7.8%) had chronic kidney diseases, and sixteen patients (15.7%) had congestive heart disease. Among the 102 enrolled patients, 30 (29.4%) were found to be infected with human adenovirus (HAdV)-7, 51 (50.0%) with HAdV-55, 4 patients (3.9%) had pneumonia caused by other types of adenoviruses, and 17 (16.7%) were detected to be infected with an adenovirus that was untypeable and lacked evidence of subtyping. Radiological findings showed bilateral interstitial consolidations in the lungs of 88 patients (86.3%) and pleural effusions in 56 patients (54.9%). (Table 1)

**Table 1:** Baseline characteristics, viral subtype, and radiographic characteristics at hospital admission of patients with HAdVs-associated pneumonia

Variable	All patients(n = 102)	Non-ARDS group(n = 61)	ARDS group(n = 41)	*P* value
**Age at admission, years**	38.0 (27.0, 54.0)	34.0 (26.0, 54.0)	41.0 (28.0, 53.0)	0.393
**Male, n (%)**	96 (94.1)	58 (95.1)	38 (92.7)	0.682
**Symptom, n (%)**				
Fever	100 (98.0)	59 (96.7)	41 (100.0)	0.514
Cough	88 (86.3)	53 (86.9)	35 (85.4)	0.827
Sputum	80 (78.4)	51 (83.6)	29 (70.7)	0.121
Dyspnea	57 (55.9)	23 (37.7)	34 (82.9)	< 0.001
Chills	46 (45.1)	21 (34.4)	25 (61.0)	0.008
Myalgia	49 (48.0)	32 (52.5)	17 (41.5)	0.276
Fatigue	54 (52.9)	32 (52.5)	22 (53.7)	0.905
Vomiting	25 (24.5)	15 (24.6)	10 (24.4)	0.982
Diarrhea	41 (40.2)	22 (36.1)	19 (46.3)	0.299
Nervous system symptoms	16 (15.7)	7 (11.5)	9 (22.0)	0.154
Dry rales	12 (11.8)	4 (6.6)	8 (19.5)	0.093
Moist rales	70 (68.6)	36 (59.0)	34 (82.9)	0.011
**Vital signs**				
Respiration rate, median (IQR), breaths per minute	21.5 (20.0, 25.0)	20.0 (20.0, 23.0)	24.0 (20.0, 28.0)	0.003
SpO_2_, median (IQR), %	95.0 (92.5, 97.0)	95.0 (90.0, 96.0)	94.0 (90.0, 96.0)	0.032
Pulse, median (IQR), beats per minute	96.5 (86.0, 108.0)	93.0 (85.0, 102.0)	104.0 (92.0, 112.0)	0.005
MAP, median (IQR), mm Hg	86.7 (74.3, 93.3)	88.0 (81.0, 93.3)	82.0 (72.0, 93.0)	0.150
PaO2:FiO2 ratio, median (IQR)	233.9 (168.0, 400.0)	317.2 (241.4, 410.0)	153.2 (97.5, 197.3)	< 0.001
**Comorbidities, n (%)**				
Hypertension	12 (11.8)	6 (9.8)	6 (14.6)	0.672
Diabetes	6 (5.9)	5 (8.2)	1 (2.4)	0.397
Chronic lung disease	9 (8.8)	6 (9.8)	3 (7.3)	0.737
Chronic liver diseases	8 (7.8)	4 (6.6)	4 (9.8)	0.711
Chronic kidney diseases	8 (7.8)	3 (4.9)	5 (12.2)	0.262
Congestive heart disease	16 (15.7)	10 (16.4)	6 (14.6)	0.811
**Viral subtype**				
HAdV-7	30 (29.4)	16 (26.2)	14 (34.1)	0.390
HAdV-55	51 (50.0)	28 (45.9)	23 (56.1)	0.313
Other types	4 (3.9)	4 (6.6)	0 (0.0)	0.147
Un-typeable	17 (16.7)	13 (21.3)	4 (9.8)	0.125
**Scores**				
SOFA score	3.0 (2.0, 5.0)	2.0 (1.0, 3.0)	5.0 (4.0, 7.0)	< 0.001
APACHE II score	7.0 (3.0, 13.0)	5.0 (2.0, 8.0)	13.0 (10.0, 20.0)	< 0.001
**Radiographic characteristics, n (%)**				
Lesions in left lung	82 (80.4)	43 (70.5)	39 (95.1)	0.002
Lesions in right lung	87 (85.3)	49 (80.3)	38 (92.7)	0.084
Lesions in bilateral lungs	67 (65.7)	32 (52.5)	35 (85.4)	0.001
Ground-glass opacity	8 (7.8)	3 (4.9)	5 (12.2)	0.262
Consolidation	88 (86.3)	50 (82.0)	38 (92.7)	0.123
Pleural effusion	56 (54.9)	26 (42.6)	30 (73.2)	0.002
**Oxygen support within 24 h of admission**				
Low flow oxygen, n (%)	37 (36.3)	22 (36.1)	15 (36.6)	0.957
High-flow nasal cannula, n (%)	15 (14.7)	5 (8.2)	10 (24.4)	0.024
Non-invasive ventilation, n (%)	17 (16.7)	4 (6.6)	13 (31.7)	0.001
Invasive ventilation, n (%)	3 (2.9)	0 (0.0)	3 (7.3)	0.062
ECMO, n (%)	0 (0.0)	0 (0.0)	0 (0.0)	
**Medication within 24 h of admission**				
Glucocorticoid, n (%)	19 (18.6)	5 (8.2)	14 (34.1)	0.001
Immunoglobulin, n (%)	22 (21.6)	9 (14.8)	13 (31.7)	0.041
Vasopressor, n (%)	3 (2.9)	0 (0.0)	3 (7.3)	0.062
CRRT, n (%)	3 (2.9)	1 (1.6)	2 (4.9)	0.563
Cidofovir, n (%)	0 (0.0)	0 (0.0)	0 (0.0)	

Data are presented as medians (IQR) and n (%). P values were calculated by Student’s t test, Mann–Whitney U test, chi-squared test or Fisher’s exact test, as appropriate. P values indicate differences between patients with or without developing HAdVs-related ARDS. A P value < 0.05 was considered statistically significant. Abbreviations: HAdVs, human adenovirus; ARDS, acute respiratory distress syndrome; IQR, interquartile range; SpO2, percutaneous oxygen saturation; PaO2, Partial pressure of oxygen; FiO2, fraction of inspired oxygen; MAP, mean arterial pressure; HAdV-7, SOFA, sepsis related organ failure assessment; APACHE, acute physiology and chronic health evaluation; human adenovirus type 7; HAdV-55, human adenovirus type 55; ECMO, extracorporeal membrane oxygenation; CRRT, Continuous renal replacement therapy

During hospitalization, 41 (40.2%) patients with adenovirus pneumonia developed ARDS and 61 (59.8%) did not. Demographic data, pre-existing conditions, and viral subtypes did not differ significantly between the patients with and without ARDS. However, compared to the non-ARDS group, patients with adenovirus-associated ARDS were more likely to present with dyspnea (P < 0.001) and chills (P = 0.008), as well as bilateral lung lesions (P = 0.001) and pleural effusions (P = 0.002) on radiological images. The adenovirus-associated ARDS group had higher respiratory (P = 0.003) and pulse rates (P = 0.005), higher SOFA (P < 0.001) and APACHE II scores (P < 0.001), and a lower SpO2 (P = 0.032) as well as a lower PaO2:FiO2 ratio (P < 0.001) than those without ARDS. Additionally, compared to patients with non-ARDS, those with ARDS were more likely to have the requirement for high-flow nasal cannula (P = 0.024), non-mechanical ventilation (P = 0.001), invasive ventilation (P = 0.062), glucocorticoid (P = 0.001) and immunoglobulin (P = 0.041) within 24 h of admission. (Table 1). Patients with adenovirus-associated ARDS had significantly higher levels of neutrophil percentage (P = 0.003), total bilirubin (P = 0.042), aspartate aminotransferase (P < 0.001), blood urea nitrogen (P = 0.001), creatinine (P = 0.008), D-dimer (P = 0.014), creatine kinase (P = 0.002), and creatine kinase isoenzyme MB (myocardial band) (P = 0.014), but lower levels of lymphocyte percentage (P = 0.001), platelet (P = 0.004), and albumin (P < 0.001) than those patients without ARDS (Table 2). (Table 2).

**Table 2:** Laboratory findings of patients with HAdV-associated pneumonia

Characteristic	Normal Range	All patients(n = 102)	Non-ARDS group(n = 61)	ARDS group(n = 41)	*P* value
Blood Routine					
White blood cell count, ×10^9^/L	3.5–9.5	5.4 (4.0, 7.7)	5.7 (4.4, 7.8)	5.0 (3.2, 7.0)	0.150
Neutrophil percentage, %	40.0–75.0	80.0 (71.2, 85.6)	77.2 (69.8, 83.4)	83.4 (77.8, 88.2)	0.003
Lymphocyte percentage, %	20.0–50.0	14.9 (9.0, 22.4)	18.4 (11.8, 24.9)	10.6 (6.9, 18.0)	0.001
Hemoglobin, g/L	115.0-150.0	134.5 (123.0, 142.0)	135.0 (125.0, 144.0)	133.0 (122.0, 141.0)	0.486
Platelet, ×10^9/L	125.0-350.0	129.5 (95.0, 177.0)	150.0 (115.0, 210.0)	102.0 (85.0, 153.0)	0.004
**Blood Biochemistry**					
Albumin, g/L	33.0–55.0	31.5 (27.9, 36.1)	34.3 (30.5, 37.7)	28.2 (25.5, 31.3)	< 0.001
Total bilirubin, µmol/L	3.0–20	9.8 (6.8, 16.8)	8.1 (6.7, 15.1)	11.8 (7.2, 20.1)	0.042
Aspartate aminotransferase, U/L	8.0–40.0	67.2 (41.6, 153.9)	57.0 (32.7, 83.0)	137.0 (61.0, 278.7)	< 0.001
Alanine aminotransferase, U/L	5.0–35.0	49.4 (28.4, 108.0)	43.0 (25.4, 108.0)	59.9 (31.1, 108.0)	0.158
Blood urea nitrogen, mmol/L	2.9–8.2	4.6 (3.2, 6.6)	4.3 (2.9, 5.5)	5.5 (4.0, 9.2)	0.001
Creatinine, µmol/L	41.0-111.0	79.5 (64.2, 97.0)	77.0 (61.0, 87.0)	86.0 (75.7, 115.9)	0.008
Lactate dehydrogenase, U/L	109.0-245.0	598.0 (365.0, 1010.0)	485.5 (261.1, 635.4)	1010.0 (675.0, 1253.0)	< 0.001
**Inflammatory Mediators**					
C-reactive protein, mg/L	0.0–8.0	91.1 (43.8, 155.6)	89.8 (39.7, 139.9)	106.8 (56.8, 180.5)	0.132
**Blood Coagulation**					
D-dimer, µg/mL	0.0-0.5	1.7 (0.6, 4.1)	1.3 (0.5, 2.8)	2.2 (0.9, 5.5)	0.014
Plasma fibrinogen, g/L	2.0–4.0	6.7 (4.5, 16.5)	6.4 (4.0, 12.2)	9.3 (5.0, 21.9)	0.066
**Myocardial Injury Mediators**					
Creatine kinase, U/L	50.0-310.0	566.2 (209.0, 1661.8)	362.8 (113.2, 867.7)	1009.6 (351.6, 2364.8)	0.002
Creatine kinase isoenzyme MB, U/L	< 24.0	25.0 (15.9, 47.5)	18.6 (12.1, 28.8)	25.9 (20.6, 53.3)	0.014

Data are presented as medians (IQR). P values were calculated by Student’s t test or Mann-Whitney U test, as appropriate. P values indicate differences between patients with or without developing HAdVs-related ARDS. A P value < 0.05 was considered statistically significant. Abbreviations: HAdVs, human adenovirus; ARDS, acute respiratory distress syndrome, IQR, interquartile range

### Medication and clinical outcomes

**Fig. 2:**
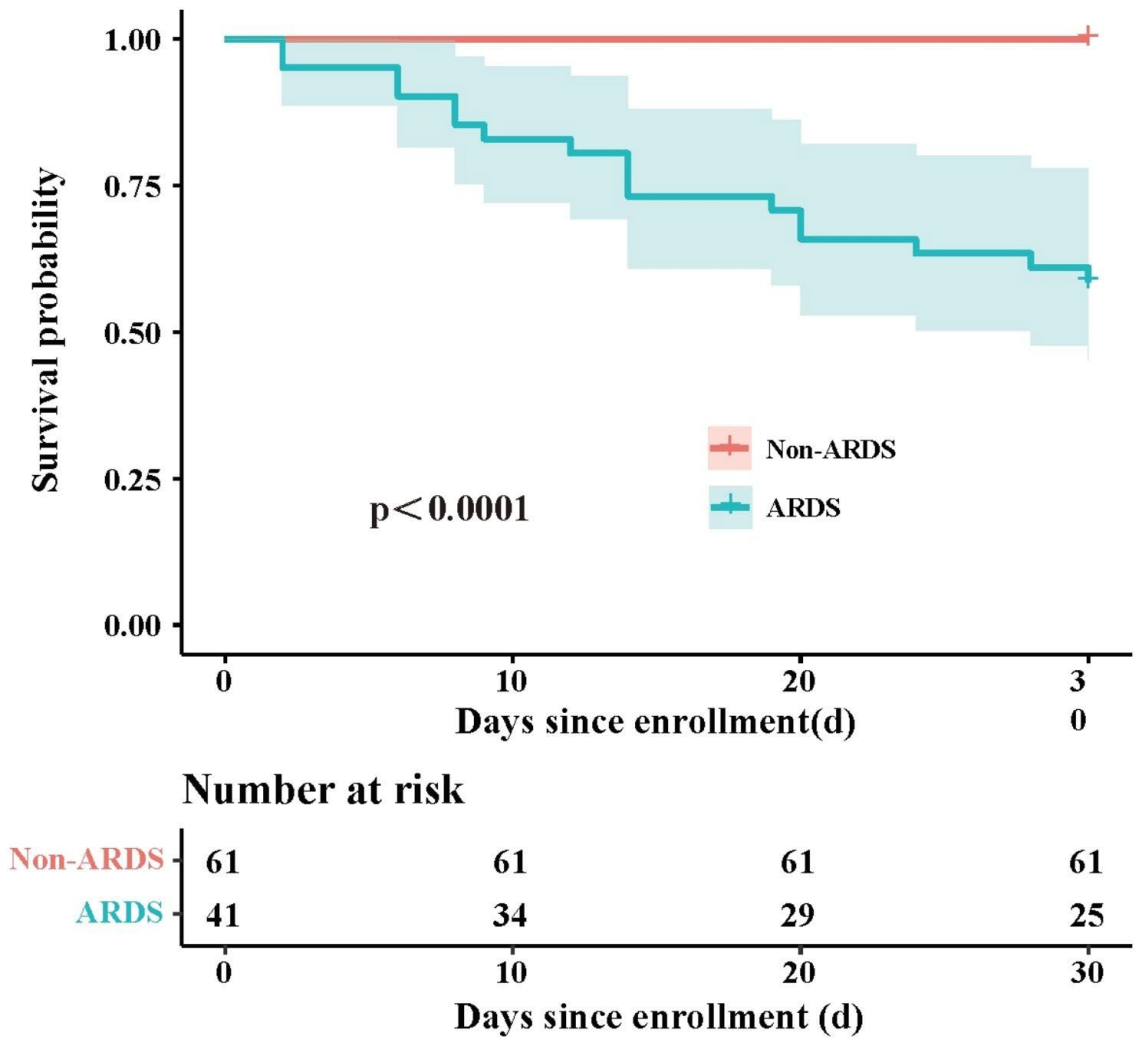
Kaplan–Meier curves of mortality for HAdVs-associated pneumonia patients with or without the development of ARDS. A log-rank test was used to evaluate differences between groups. Abbreviations: HAdVs, human adenovirus; ARDS, acute respiratory distress syndrome

Overall, the in-hospital mortality was 16.7% among patients diagnosed with adenovirus pneumonia who constituted our cohort, with higher mortality in patients with adenovirus-associated ARDS than in those without (41.5% vs. 0.0%; P < 0.001) (Table 3). The Kaplan–Meier survival curve demonstrated a significant survival benefit among adenovirus patients without ARDS compared to patients with adenovirus-associated ARDS (P < 0.001) (Fig. 2) that all the mortality was seen in patients with ARDS and none in those without. Regarding respiratory support, adenovirus-associated ARDS patients were more likely to require and receive invasive mechanical ventilation and extracorporeal membrane oxygenation (ECMO) support (P < 0.05) (Table 3). More patients with adenovirus-associated ARDS were treated with glucocorticoids, immunoglobulins, vasopressors, continuous renal replacement therapy (CRRT), and cidofovir (16/41) than those without. Patients with adenovirus-associated ARDS were also more likely to develop septic shock, acute cardiac injury, and acute kidney injury, and require admission to the ICU (P < 0.05) (Table 3).

**Table 3:** Treatments and outcomes of patients with HAdV-associated pneumonia

	All patients(n = 102)	Non-ARDS group(n = 61)	ARDS group(n = 41)	*P* value
Oxygen support				
Low flow oxygen, n (%)	31 (30.4)	31 (50.8)	0 (0.0)	< 0.001
High-flow nasal cannula, n (%)	9 (8.8)	5 (8.2)	4 (9.8)	1.000
Non-invasive ventilation, n (%)	4 (3.9)	2 (3.3)	2 (4.9)	1.000
Invasive ventilation, n (%)	31 (30.4)	3 (4.9)	28 (68.3)	< 0.001
ECMO, n (%)	7 (6.9)	0 (0.0)	7 (17.1)	0.001
**Medication**				
Glucocorticoid, n (%)	48 (47.1)	12 (19.7)	36 (87.8)	< 0.001
Immunoglobulin, n (%)	42 (41.2)	12 (19.7)	30 (73.2)	< 0.001
Vasopressor, n (%)	29 (28.4)	0 (0.0)	29 (70.7)	< 0.001
CRRT, n (%)	13 (12.7)	2 (3.3)	11 (26.8)	< 0.001
Cidofovir, n (%)	20 (19.6)	4 (6.6)	16 (39.0)	< 0.001
**Complication**				
Acute cardiac injury, n (%)	48 (47.1)	16 (26.2)	32 (78.0)	< 0.001
Acute kidney injury, n (%)	24 (23.5)	4 (6.6)	20 (48.8)	< 0.001
**Clinical outcomes**				
Hospital mortality, n (%)	17 (16.7)	0 (0.0)	17 (41.5)	< 0.001
ICU admission, n (%)	61 (59.8)	22 (36.1)	41 (100.0)	< 0.001
Length of hospital stay of survivors, days	11.0 (9.0, 16.0)	10.0 (8.0, 15.0)	16.0 (11.5, 19.0)	0.006

Data are presented as medians (IQR) and n (%). P values were calculated by Student’s t test, Mann–Whitney U test, chi-squared test or Fisher’s exact test, as appropriate. P values indicate differences between patients with or without developing HAdVs-related ARDS. A P value < 0.05 was considered statistically significant. Abbreviations: HAdVs, human adenovirus; ARDS, acute respiratory distress syndrome; IQR, interquartile range; ECMO, extracorporeal membrane oxygenation; CRRT, Continuous renal replacement therapy; ICU; intensive care unit

### Model development for differentiating adenovirus-associated ARDS from adenovirus Pneumonia patients without ARDS

Univariate and multivariate logistic regression analyses are illustrated in Table 4. Multivariate logistic regression models revealed that exhibit dyspnea symptoms (OR:4.191, 95% CI:1.234–14.241, p = 0.022), higher SOFA scores (OR:1.370, 95% CI:1.072–1.751, p = 0.012), higher LDH (lactate dehydrogenase) levels (OR:1.002, 95% CI:1.000–1.003, p = 0.026), and the use of non-invasive ventilation or invasive ventilation (OR:5.007, 95% CI:1.220–20.551, p = 0.025) were significant predictors of adenovirus-associated ARDS. (Table 4).

**Table 4:** Univariable and multivariable analysis of Independent Risk Factors for developing ARDS in patients with HAdV-associated pneumonia

Variable	Univariate logistic analysis		Multivariate logistic analysis	
	OR (95%CI)	*P*-value	OR (95%CI)	*P*-value
Dyspnea	8.025 (3.059 ~ 21.049)	< 0.001	4.191 (1.234 ~ 14.241)	0.022
Chills	2.976 (1.311 ~ 6.759)	0.009		
Moist rales	3.373 (1.291 ~ 8.812)	0.013		
Respiration rate	1.116 (1.028 ~ 1.212)	0.009		
SpO_2_	0.866 (0.769 ~ 0.975)	0.017		
Pulse	1.040 (1.012 ~ 1.068)	0.005		
PaO2/FiO2	0.979 (0.970 ~ 0.987)	< 0.001		
SOFA	1.697 (1.348 ~ 2.138)	< 0.001	1.370 (1.072 ~ 1.751)	0.012
APACHE II	1.255 (1.144 ~ 1.377)	< 0.001		
Lesions in left lung	8.163 (1.779 ~ 37.464)	0.007		
Lesions in bilateral lungs	5.286 (1.942 ~ 14.388)	0.001		
Pleural effusion	3.671 (1.558 ~ 8.652)	0.003		
Neutrophil percentage	1.033 (0.995 ~ 1.072)	0.086		
Lymphocyte percentage	0.926 (0.879 ~ 0.974)	0.003		
Platelet	0.992 (0.986 ~ 0.998)	0.014		
Albumin	0.807 (0.730 ~ 0.893)	< 0.001		
Total bilirubin	1.057 (1.009 ~ 1.106)	0.019		
Aspartate aminotransferase	1.008 (1.003 ~ 1.013)	0.001		
Blood urea nitrogen	1.173 (1.024 ~ 1.344)	0.021		
Creatinine	1.002 (0.997 ~ 1.007)	0.407		
Lactate dehydrogenase	1.003 (1.001 ~ 1.004)	< 0.001	1.002 (1.000 ~ 1.003)	0.026
D-dimer	0.999 (0.998 ~ 1.001)	0.394		
Creatine kinase	1.000 (1.000 ~ 1.001)	0.016		
Creatine kinase isoenzyme MB	1.014 (0.995 ~ 1.033)	0.141		
High-flow nasal cannula	3.613 (1.133 ~ 11.522)	0.030		
Non-invasive ventilation or Invasive ventilation	9.120 (2.768 ~ 30.046)	< 0.001	5.007 (1.220 ~ 20.551)	0.025
Glucocorticoid	5.807 (1.896 ~ 17.792)	0.002		
Immunoglobulin	2.683 (1.021 ~ 7.049)	0.045		

A P value < 0.05 was considered statistically significant. Abbreviations: HAdVs, human adenovirus; ARDS, acute respiratory distress syndrome; OR, odd ratios; CI, confidence interval; SpO2, percutaneous oxygen saturation; PaO2, Partial pressure of oxygen; FiO2, fraction of inspired oxygen; SOFA, sepsis related organ failure assessment; APACHE, acute physiology and chronic health evaluation

### Nomogram establishment and performance

**Fig. 3:**
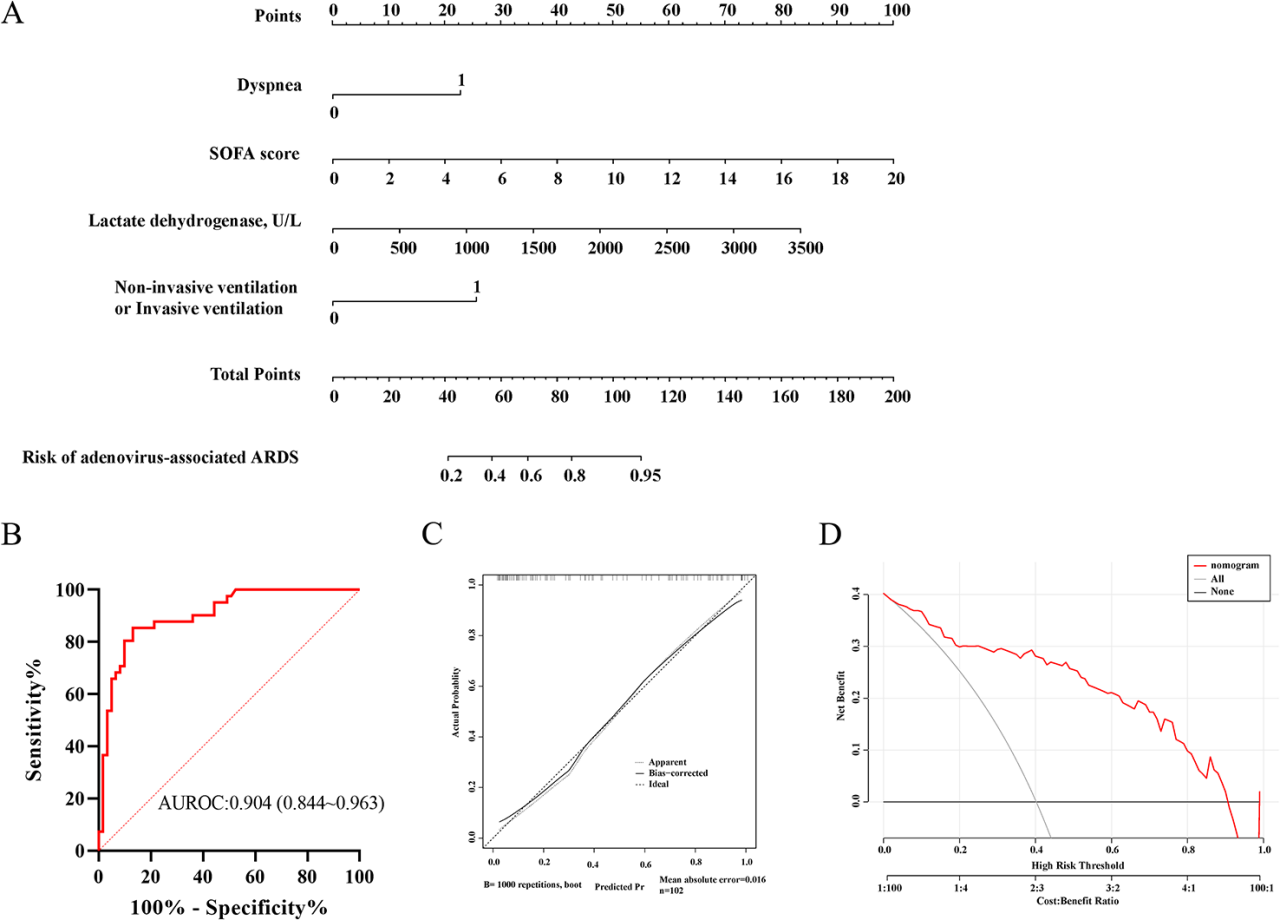
Construction of nomogram for early prediction of HAdVs-related ARDS. (**A**) Developed ARDS prediction nomogram in HAdVs-associated pneumonia patients. (**B**)The ROC for the performance of the ARDS prediction nomogram. (**C**) Calibration curves of the ARDS nomogram prediction in the HAdVs-associated pneumonia trial

Multivariate logistic regression showed that dyspnea as well as higher SOFA scores, higher LDH levels and non-invasive ventilation or invasive ventilation were risk factors for adenovirus-associated ARDS. A nomogram was constructed to visualize the predicted model, as shown in Fig. 3A. It demonstrated good accuracy in differentiating adenovirus-associated ARDS from adenovirus pneumonia in patients without ARDS, with a C-statistic of 0.904 (95% CI 0.844–0.963). The predictive ability was assessed using receiver operating characteristic (ROC) curve analysis (Fig. 3B), and the area under the ROC curve (AUROC) was 0.904. The calibration curves in Fig. 3C show that the predictive curves were close to the ideal curve, demonstrating excellent calibration of the nomogram. The results of the DCA showed good benefits in clinical practice of using the nomogram in predicting adenovirus pneumonia-associated ARDS. (Fig. 3D).

## Discussion

In this study, we observed that adenovirus pneumonia complicated by ARDS had a high mortality rate (41.5%). To our knowledge, this is the first retrospective analysis to compare the risk factors for ARDS incidence among patients with adenovirus pneumonia. We found that dyspnea, higher SOFA scores, higher LDH levels and the requirement for non-invasive or invasive ventilation correlated with a higher risk of developing ARDS in patients with adenovirus-associated pneumonia. Additionally, we developed a nomogram with good accuracy in the early prediction of ARDS in patients with adenovirus-associated pneumonia.

The epidemiological trends of adenovirus infections in immunocompetent patients in Central South China in recent years were similar to those reported previously [2, 30–33]. In this study, we observed that young and middle-aged male patients comprised the majority of the study population, and flu-like symptoms, such as fever, cough, and sputum, were the most frequent clinical manifestation at the onset of illness. Most patients had no underlying disease. In addition, HAdV-7 and HAdV-55 were the main epidemic strains among patients with HAdV-related ARDS in recent years, similar to the epidemic strains reported in severe adenovirus-related pneumonia in immunocompetent patients in Asia in recent years [2, 27, 34–38].

Our results showed that ARDS is a serious complication in patients with HAdV-related pneumonia. Compared with the occurrence of mortality in ARDS caused by various etiologies (ranging from 35 to 50%) [39], the mortality in HAdV-associated ARDS in our study was at a high level of 41.5%. In the HAdV-associated ARDS group, the SOFA score, a commonly clinically elevated score used to evaluate organ dysfunction in critically ill patients, was significantly higher and was found to be independently associated with the risk of ARDS development, suggesting a more severe disease state in patients with HAdV-associated ARDS at admission. This highlights the importance of early recognition of the risk of ARDS.

We found that have dyspnea and the requirement for non-invasive ventilation or invasive ventilation were risk factors for the development of ARDS among patients with HAdV-associated pneumonia. Moreover, a lower oxygenation index and SPO2 were found in patients with ARDS development. Compared with non-ARDS patients, patients with adenovirus-related ARDS are more likely to have double-lung consolidations, which is also correlated with poor respiratory function. Furthermore, dyspnea in adult immunocompetent patients with HAdV-associated ARDS has been previously reported; however, only in case reports [7, 16, 40]. Overall, we suggest that evidence of a gradual worsening of dyspnea may reflect early disease progression of HAdV-associated ARDS.

In terms of laboratory results, HAdVs associated with ARDS showed unfavorable levels of biomarkers of hepatic injury (elevated transaminase and total bilirubin, decreased albumin), cardiac injury (elevated creatine kinase and creatine kinase isoenzyme MB), and kidney injury (elevated creatinine and blood urea nitrogen). Furthermore, the data on complications gathered in this study, together with the previous cases [8, 41, 42], confirm that HAdV-associated ARDS that occurred along with one or more organ injuries (including liver, kidney, and cardiac) was common in adult immunocompetent patients. The reasons for this are multifactorial, and the specific mechanisms remain unclear. We speculate that there could be three reasons for this: (i) increased virulence and pathogenicity or altered tissue tropism due to HAdV mutation or recombination [43, 44], (ii) co-infections due to hospital setting or invasive management, and (iii) overexposure to hepatotoxic drugs, nephrotoxic drugs, or cardiotoxic drugs during treatment. Furthermore, LDH, a general indicator of tissue damage found in almost all body cells [45, 46], was higher in patients who showed progression to ARDS than in those who did not. More importantly, we found that elevated LDH levels were associated with a greater risk of developing HAdV-associated ARDS, suggesting a possible relationship between tissue damage and incidence of HAdV-associated ARDS.

Previous investigators developed multiple prediction models that contained different risk factors to identify patients at risk for ARDS in a setting of sepsis [21], COVID-19 [47] and severe acute pancreatitis [20], reflecting that variations in models can exist within the various ARDS populations. Our study focused on patients with HAdV-associated pneumonia, which is more specific and makes the HAdV-related ARDS prediction model more targeted. In our study, the clinical data of 41 patients with HAdV-associated ARDS and 61 patients with HAdV-associated pneumonia were represented, and the clinical characteristics of the two groups were compared and analyzed. Multivariate logistic regression indicated that dyspnea, SOFA score, LDH levels and mechanical ventilation status were risk factors for HAdV-associated ARDS. Based on these four risk factors, we developed an effective nomogram model for the early prediction of HAdV-associated ARDS. This model demonstrated good performance in predicting ARDS among patients with HAdV-associated pneumonia, with a good C-statistic of 0.904. The calibration curves show that the predictive curves were close to the ideal curve, demonstrating excellent calibration of the nomogram. The DCA curve demonstrated that this model could reliablely predict the risk of developing ARDS in HAdV-associated pneumonia patients with a superior net benefit across a broad range of threshold probabilities. Our study found that adenovirus-associated ARDS can cause more organ injuries (including liver, kidney, and cardiac) and lead to higher mortality, so it is of great clinical significance to predict the progression of ARDS in patients with adenovirus pneumonia and give individualized intervention. At present, there is still a lack of an early, simple, and effective prediction model, which may lead to a poor prognosis for patients with adenoviral pneumonia. The nomogram we have established combines the readily and early available clinical symptoms and laboratory results to reasonably predict HAdV-associated ARDS. Therefore, the nomogram would be easier to use and promote at all hospital levels.

### Strengths and limitations

To our knowledge, the present study is the largest to investigate HAdV-associated ARDS and evaluate the predictors of developing ARDS among patients with HAdV-associated pneumonia. As this was a retrospective study, it had certain limitations associated with this type of study. An additional limitation is the absence of external validation. Although the nomogram can predict the early development of ARDS from HAdV-associated pneumonia, large-sample prospective cohort validation studies are needed to verify the clinical benefit of the nomogram. Although the genetic analysis of HAdV strains circulating in different parts of China showed that the genomes of the HAdV strains circulating in China have remained relatively stable over time and geographic space [15, 43], continuous surveillance of HAdV changes and further study on the virulence and pathogenicity of the virus strain in immunocompetent adults with or without ARDS is necessary.

## Conclusion

Our study established a prediction model incorporating dyspnea, higher SOFA scores, higher LDH levels and and the requirement for non-invasive or invasive ventilation to predict ARDS risk in HAdV-associated pneumonia in immunocompetent patients. However, the findings of this study should be validated in a subsequent prospective study.

## Data Availability

The datasets used and/or analyzed during the current study are available from the corresponding author (Pinhua Pan,Phone: +86-0731-89753287, E-mail: pinhuapan668@csu.edu.cn) on reasonable request.
